# Hare-to-Human Transmission of Francisella tularensis subsp. holarctica, Germany

**DOI:** 10.3201/eid2101.131837

**Published:** 2015-01

**Authors:** Peter Otto, Rebekka Kohlmann, Wolfgang Müller, Sandra Julich, Gabriele Geis, Sören G. Gatermann, Martin Peters, Peter Johannes Wolf, Edvin Karlsson, Mats Forsman, Kerstin Myrtennäs, Herbert Tomaso

**Affiliations:** Friedrich-Loeffler-Institut, Federal Research Institute for Animal Health, Jena, Germany (P. Otto, W. Müller, S. Julich, H. Tomaso);; Institute of Medical Laboratory Diagnostics Bochum, Bochum, Germany (R. Kohlmann, G. Geis);; Ruhr-University, Bochum (S.G. Gatermann);; National Veterinary Laboratory Arnsberg, Arnsberg, Germany (M. Peters);; Evangelisches Krankenhaus, Lippstadt, Germany (P.J. Wolf);; Swedish Defence Research Agency, Umea, Sweden (E. Karlsson, M. Forsman, K. Myrtennäs)

**Keywords:** Tularemia, Francisella, Francisella tularensis subsp. *holarctica*, hare, European brown hare, Lepus europaeus, zoonoses, North Rhine-Westphalia, Germany

## Abstract

In November 2012, a group of 7 persons who participated in a hare hunt in North Rhine-Westphalia, Germany, acquired tularemia. Two *F. tularensis* subsp. *holarctica* isolates were cultivated from human and hare biopsy material. Both isolates belonged to the FTN002–00 genetic subclade (derived for single nucleotide polymorphisms B.10 and B.18), thus indicating likely hare-to-human transmission.

Tularemia is a zoonotic disease caused by the gram-negative bacterium *Francisella* *tularensis* (*1*). Currently, there are 4 validly published subspecies. *F. tularensis* subsp. *tularensis* is the most virulent subspecies and occurs only in North America. *F. tularensis* subsp. *holarctica* is less virulent and occurs throughout the Northern hemisphere. *F. tularensis* subsp. *mediasiatica* was isolated in central Asia, and *F. tularensis* subsp. *novicida*, which has low virulence in humans, seems to be distributed globally (*2*).

Various PCR-based assays have been established for the detection of *F. tularensis* or for the diagnosis of tularemia. An accurate population structure has been defined by using single nucleotide polymorphisms (SNPs) and insertion/deletion mutations (INDELs) with potential canonical properties. Currently, this population is divided into 4 major genetic clades: B.4, B.6, B.12, and B.16 (*3**–**6*). The taxonomic nomenclature of major clades in *F. tularensis* subsp. *holarctica* is based on clade-specific canonical SNP markers (*3**,**4*)*.* In Europe, the strains of clades B.12 and B.6 dominate (*6*). The latter is found particularly in large areas in northern, western, and central Europe, including Germany (*5**–**9*).

## The Study

On November 2, 2012, 15 European brown hares (*Lepus europaeus*) were shot during a hunt in Rüthen-Meiste, district Soest in the federal state of North Rhine-Westphalia, Germany (Figure). The animals seemed healthy and showed normal escape behavior. Upon inspection, the animals that had been shot showed no signs of disease. Consequently, all animals were skinned, eviscerated, and dissected. Portioning of the hares was done 2 days later. Within a few days, 7 healthy persons who had contact with the hare carcasses showed varied symptoms of illness. Tularemia was suspected because of the signs and symptoms in combination with exposure in a tularemia-endemic area. Exposure, clinical symptoms, and time of onset of symptoms of all patients (A to G) are described in the Table. All patients were treated successfully with doxycycline.

**Figure:**
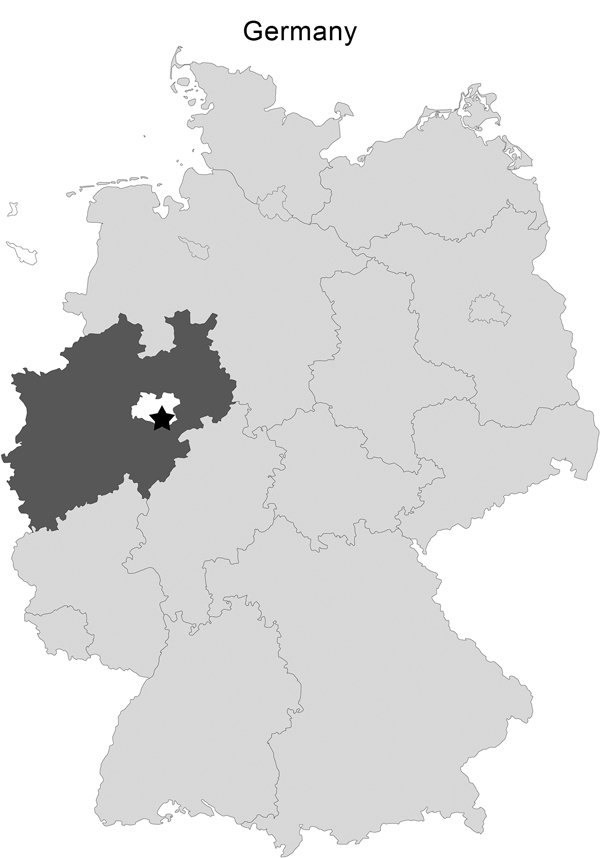
Area of Germany where hares were hunted on November 2, 2012: Rüthen-Meiste (black star; latitude 51.512890, longitude 8.487493, altitude 380 m), Soest district (white) of the federal state of North Rhine-Westphalia (dark gray).

**Table:** Exposure, clinical presentation, and time of onset of clinical symptoms of tularemia in patients, Germany, 2012

Patient	Exposure type	Clinical symptoms	Symptom onset
A	Skinning	Fever, cold, cough	Nov 12
B	Skinning	Chills, fever, joint pain	Nov 9
C	Dissection	Fever, nodes and skin ulcers	Nov 4
D	Cut up carcasses	Fever, cold, cough, joint pain, convulsions	Nov 4
E	Cut up carcasses	Cough, pleural effusion, weight loss	Unknown
F	Portioning of hares	Fever, skin ulcers, lymphadenopathy	Unknown
G	Portioning of hares	Fever, skin ulcers, weight loss	Unknown

Human serum samples were collected about 5 weeks after infection from patients C and E and pleural fluid was obtained from patient E. On day 2 of incubation, the human isolate (12T0062) showed small pale-white to gray colonies on Columbia blood agar and chocolate agar, whereas no growth occurred on MacConkey agar plates. Ten organ specimens (from aorta, back and thigh muscles, lymph nodes, spinal cord) from 4 of the hares handled by the patients were tested for *Francisella* spp. in the National Reference Laboratory for Tularemia at the Friedrich-Loeffler-Institut in Jena, Germany. From all hare organs, only a single *Francisella* sp. was isolated from a spinal cord sample (13T0009) on cysteine heart agar Becton Dickinson GmbH, Heidelberg, Germany), which contains antibiotics. The strains were susceptible to erythromycin with inhibition zones between 22 and 24 mm corresponding to biovar I. Details of further methods that have been applied in the study are shown in the Technical Appendix.

Both samples were identified as *F. tularensis* subsp. *holarctica* of clade B.6. For B.19, the SNP was C; results of Ftind33 and Ftind38 assays were IN, and the result of the Ftind49 assay was DEL. Both samples had T for SNP B.7, G for B.10 and T for B.18. Therefore, the strains were considered derived from SNPs B.10 and B.18. Blood serum samples of patients C and E were positive for *F. tularensis* with very high values of the optical density in the ELISA, 2.886 and 3.121, respectively.

## Conclusions

The re-emergence of tularemia in Germany has been described in previous studies (*10*). Infected hares are believed to be the sources of most cases of tularemia in Germany. However, to our knowledge, route of transmission has not been demonstrated by isolation and genotyping of the pathogen from the suspected source and the patient (*11**–**13*). In this study, we therefore described not only the clinical and epidemiologic data and the laboratory diagnostic findings for determining tularemia, but also the results of genotyping the *Francisella* spp. isolated from epidemiologically linked hares and humans.

All 7 infected persons (A to G) in this outbreak showed influenza-like symptoms of varying intensity (Table), but symptoms were also related to the route of infection. The 5 patients (A to E) who had fever as well as respiratory and topical symptoms were exposed to aerosols and had direct skin contact when skinning and processing hare carcasses. The 2 patients (F and G) who portioned the meat had lesions on their hands, enlarged lymph nodes, and fever.

The isolated *F. tularensis* subsp. *holarctica* strains were susceptible to erythromycin and thus belong to the *F. tularensis* subsp. *holarctica* biovar I group. Because of the inability of the duplex PCR assay to distinguish between *F. tularensis* subsp. *holarctica* strains (*8*), we performed a combined SNP and INDEL analysis using real-time PCR. Here, we were able to isolate *F. tularensis* subsp. *holarctica* biovar I strains from a hare and a human; both isolates could be assigned to the genetic clade B.6 in the first order of discrimination [B.19(C), Ftind33(IN), Ftind38(IN), and Ftind49(DEL)]. The isolates also showed an identical genotyping profile for B.7(T), B.10(G), B.18(T) in the second order of discrimination, which corresponds with a previously described subclade represented by the strain FTNF002–00 that was isolated from a patient from France who had bacteremia (*3**,**4*). Thus, the genetic subtyping results are consistent with the proposed transmission route of the epidemiologically linked (hare–human transmission) *F*. *tularensis* subsp., since both belonged to the same genetic subclade.

The current phylogeography of *F. tularensis* subsp. *holarctica* revealed that 2 major groups of virulent strains exist in Europe (*5*). In the western European countries of Spain, France, Switzerland, and Italy, strains of the FTNF002–00 group dominate, whereas strains of clade B.12 seem to predominate in eastern and northern Europe as reported from Austria, Czech Republic, Finland, Georgia, Hungary, Romania, Russia, Slovakia, Sweden, and Ukraine (*4**,**5**,**8**,**13*). Vogler et al. (*4*) suggest that it is likely that the spread of strains in subclade FTNF002–00 throughout France and the Iberian Peninsula was a very recent event. In Germany, isolates of both groups have been identified and a sharp dividing line in terms of occurrence of the clades B.12 and B.6 from the northwest to the southeast of the country has been shown (*8*). The reasons for this are not known; possible causes could be environmental and epidemiologic differences. Alternately, a mixture of both genetic clades and biovars have been reported in Bulgaria, Kazakhstan, Norway, Russia, and Sweden (*7**,**14**,**15*).

The genome of *F. tularensis* subsp. *holarctica* is highly conserved and strains can hardly be discriminated. Therefore, the discriminatory power of the applied assays is limited and other field isolates from this area may show identical characteristics (H. Tomaso, unpub. data). For epidemiologic and forensic purposes, whole-genome sequencing of a multitude of strains from well-documented outbreaks and the surrounding areas should be performed to clarify and possibly quantify the genetic changes that can finally confirm or rule out the route of transmission.

Technical Appendix Details of methods applied to the process of characterizing and identifying *Francisella* subsp. isolated from human and hare organ samples.
