# Supplementary material for: Hare-to-Human Transmission of Francisella tularensis subsp. holarctica, Germany
Source: Emerg Infect Dis. 2015 Jan;21(1):153–5. doi: 10.3201/eid2101.131837 (PMC4285259; doi:10.3201/eid2101.131837)
Supplement: Technical Appendix — Details of methods applied to the process of characterizing and identifying Francisella subsp. isolated from human and hare organ samples. [file 13-1837-Techapp-s1.pdf]

Article DOI: http://dx.doi.org/10.3201/eid2101.131837

Hare-to-Human Transmission of *Francisella tularensis* subsp. *holarctica*, Germany

Material and Methods

Cultivation

The organ samples were streaked on modified Martin-Lewis-agar (BD Biosiences, Heidelberg, Germany) and cysteine heart agar (CHA; Becton Dickinson GmbH, Heidelberg, Germany) that was supplemented with 10% chocolatised sheep blood and was prepared with and without antibiotics. The selective CHA contained 100 mg ampicillin (Sigma-Aldrich Chemie, Taufkirchen, Germany) and 600,000 U polymyxin B (Sigma-Aldrich Chemie) per one liter of culture medium. All plates were incubated at 37°C with 5% CO_2 for up to 8 days.

Erythromycin Susceptibility

The isolates were tested for their erythromycin susceptibility by using erythromycin discs (30 µg; Oxoid, Wesel, Germany) according to the manufacturer's instructions to discriminate the susceptible *F. tularensis* subsp. *holarctica* biovar I from the resistant biovar II.

DNA Extraction

50 mg of organ material were lysed and the DNA was extracted by using the High Pure PCR Template Preparation Kit (Roche Diagnostics, Mannheim, Germany) according to the manufacturer`s instructions. Colonies were suspended in 200 µL phosphate-buffered saline, boiled at 95°C for 10 minutes and DNA was prepared as described above. Finally, DNA was eluted in 200 µL elution buffer. Five µL were applied in duplex PCR assay.

Duplex PCR Assay

A duplex PCR targeting the locus FtM-19 that distinguishes the two major subspecies *F. tularensis* subsp. *holarctica* and *F. tularensis* subsp. *tularensis* based on the 30 bp-deletion was carried out as described by Johansson et al. (*1*).

Single Nucleotide Polymorphism and Insertion Deletion Mutants Discrimination Assay

Four loci (B.19, Ftind33, Ftind38 and Ftind49) described by Svensson et al. (*2*) have been found to be useful for identification of the genetic clades B.4, B.6 and B.12 of *Francisella tularensis* subsp. *holarctica*. Further single nucleotide polymorphisms (SNPs), B.7, B.10, (*3*) and B.18 (*2*) were selected for discrimination of clade B.6 into subclades. SNP and INDEL determination was carried out in duplicates by using real-time PCR assays with SYBR Green as described in Svensson *et al*. (*2*), with a few modifications. The reaction mixture consisted of 10 μL 2x LightCycler 480 SYBR Green I Master (Roche Diagnostics GmbH, Mannheim, Germany), 0.8 μL of each 10 μM primer (TIB MOLBIOL Syntheselabor GmbH, Berlin, Germany), 2 μL sample DNA adjusted to 500 pg/ μL, and deionized water to a total volume of 20 μL. Each sample was amplified by using a mastermix containing either the derived or the ancestral primer as forward primers and the common reverse primer. Temperature time profile: 2 min at 50°C, 10 min at 95°C, 41 cycles [15 s at 95°C, 1 min at 60°C], 1 min at 95°C, 1 min at 20°C, then continuous heating to 95°C for melting curve analysis (heating rate: 0.11°C/s). Primer sequences are listed in the Technical Appendix Table.

The SNP status was determined by inspecting the amplification curves. Amplification appeared earlier in reaction mixtures containing the forward primers with a matching base. A positive result was assigned for the ancestral or derived assay with the lower Cq value, when there was a minimum difference of ΔCq $\geq$2.

In case of a deletion (DEL), the Cq values belonging to the OUT primer were lower than for the IN primer. In case of an insertion (IN), both Cq values were almost similar with a ΔCq <2.

Enzyme-linked Immunosorbent Assay

The ELISA for detection of *Francisella* antibodies was performed as published by Porsch-Ozcürümez et al. (*4*), but with some modifications (*5*).

References

1. Johansson A, Ibrahim A, Göransson I, Eriksson U, Gurycova D, Clarridge JE III, et al. Evaluation of PCR-based methods for discrimination of *Francisella* species and subspecies and development of

a specific PCR that distinguishes the two major subspecies of *Francisella tularensis*. J Clin Microbiol. 2000;38:4180–5. PubMed

2. Svensson K, Granberg M, Karlsson L, Neubauerova V, Forsman M, Johannson A. A real-time PCR assay for hierarchical identification of *Francisella* isolates. PLoS ONE. 2009a;4:e8360. PubMed http://dx.doi.org/10.1371/journal.pone.0008360

3. Vogler AJ, Birdsell D, Price LB, Bowers JR, Beckstrom-Sternberg SM, Auerbach RK, et al. Phylogeography of *Francisella tularensis*: global expansion of a highly fit clone. J Bacteriol. 2009b;191:2474–84. PubMed http://dx.doi.org/10.1128/JB.01786-08

4. Porsch-Ozcürümez M, Kischel N, Priebe H, Splettstosser W, Finke E-J, Grunow R. Comparison of enzyme-linked immunosorbent assay, Western blotting, microagglutination, indirect immunofluorescence assay, and flow cytometry for serological diagnosis of tularemia. Clin Diagn Lab Immunol. 2004;11:1008–15. PubMed

5. Chaignat V, Djordjevic-Spasic M, Ruettger A, Otto P, Klimpel D, Müller W, et al. Evaluation of seven serological assays for diagnosis of tularemia. BMC Infect Dis. 2014;14:234. PubMed http://dx.doi.org/10.1186/1471-2334-14-234

Technical Appendix Table: Canonical SNP and insertion/deletion mutations were detected by using a common reverse primer and primers specific for the ancestral or derived state (*2*,*3*)

Marker	SNP state	Primer	Sequence 5´→3´
B.19	A	Derived	gcgggcTTGCTACTGATGGTTTAACTa
	C	Ancestral	gcgggcagggcggcTTGCTACTGATGGTTTAACTc
		Common	CAATACGTCACTTATGCAGTGAT
Ftind33		IN	TCTAAATTTAAGCAATGTTTCTAACT
		OUT	ATCATCGTATAAGAAATCAACTT
		CP	TCAACCTTACAGAATAAGAATGT
Ftind38		IN	ACCCAATAAGCTCACCATCA
		OUT	ATCTTTCTCAGGTACAGACTTTA
		CP	AGTACTATTTGCTTATCCAAGTGAA
Ftind49		IN	AAGATTAAGTGGCAATTTAC
		OUT	TTCAACCTGGACAACCACTA
		CP	AGGATCCCAGTTAGGTTTAGTA
B.7	C	Derived	cggggcggggcggggcggggGTTTTGCAGCTAATAATTTCATTGgC
	T	Ancestral	ttttttttttttttttttttTGGTTTTGCAGCTAATAATTTCATTGgT
		Common	GTCCTTGTTAGTCAAAGCGCTATAA
B.10	G	Derived	cggggcggggcggggcggggGCCATCACTAGTAAATACCACATTAAaC
	A	Ancestral	ttttttttttttttttttttTGCCATCACTAGTAAATACCACATTAAgT
		Common	TTGTAATATTAGCTMGAAAAGTAGATGAA
B.18	T	Derived	gcgggcAGCAGCAGGACAAATAGt
	C	Ancestral	gcgggcagggcggcAGCAGCAGGACAAATAGc
		Common	TTGTGTCGATTCAAAACCAGACTTA
